# Morphological and Compositional Analysis on Thermal Deposition of Supercritical Aviation Kerosene in Micro Channels

**DOI:** 10.3390/molecules28114508

**Published:** 2023-06-01

**Authors:** Ao Sun, Cui Ye, Chenyang Yao, Lifeng Zhang, Ji Mi, Wenjun Fang

**Affiliations:** 1 School of Petroleum Engineering, Northeast Petroleum University, Daqing 163318, China; sunaonepu@hotmail.com; 2 Department of Chemistry, Zhejiang University, Hangzhou 310058, China; 22037070@zju.edu.cn (C.Y.); 22037068@zju.edu.cn (C.Y.); 3 CenerTech Tianjin Chemical Research and Design Institute Co., Ltd., Tianjin 300131, China; zlifeng@zju.edu.cn; 4 Center of Chemistry for Frontier Technologies, Zhejiang University, Hangzhou 310058, China

**Keywords:** RP-3, Raman spectroscopy, supercritical, thermal oxidation, thermal deposition

## Abstract

The integration of active cooling systems in super or hypersonic aircraft using endothermic hydrocarbon fuels is considered an effective way to relieve the thermal management issues caused by overheating. When the temperature of aviation kerosene exceeds 150 °C, the oxidation reaction of fuel is accelerated, forming insoluble deposits that could cause safety hazards. This work investigates the deposition characteristic as well as the morphology of the deposits formed by thermal-stressed Chinese RP-3 aviation kerosene. A microchannel heat transfer simulation device is used to simulate the heat transfer process of aviation kerosene under various conditions. The temperature distribution of the reaction tube was monitored by an infrared thermal camera. The properties and morphology of the deposition were analyzed by scanning electron microscopy and Raman spectroscopy. The mass of the deposits was measured using the temperature-programmed oxidation method. It is observed that the deposition of RP-3 is highly related to dissolved oxygen content (DOC) and temperature. When the outlet temperature increased to 527 °C, the fuel underwent violent cracking reactions, and the structure and morphology of deposition were significantly different from those caused by oxidation. Specifically, this study reveals that the structure of the deposits caused by short-to-medium term oxidation are dense, which is different from long-term oxidative deposits.

## 1. Introduction

With the development of aeroengine technology, the increasing speed of aircraft has dramatically increased the thermal load on the aircraft body and engines [1]. Cooling with the fuel carried by the aircraft can effectively solve the problem of the heat load of the aircraft [2]. Because this method uses fuel for cooling, it carries part of the heat from the fuselage to the combustion chamber by energy transfer, so the technique is also known as regenerative cooling [3]. Regenerative cooling can cool down high-temperature components in the aircraft by using the heat capacity and endothermic chemical reactions of aviation kerosenes. At the same time, the fuel itself undergoes thermochemical reactions that generate small-molecule products. Fuel carrying small-molecule products is not only easier to burn when it enters the combustion chamber, but also releases the heat absorbed during the cracking reaction, which improves the energy utilization rate [4]. While absorbing heat, aviation kerosene will also be produced in a high-temperature environment in addition to the presence of cracking reactions including oxidation, polymerization [5,6,7], and other complex reactions. This can easily form deposits in the fine pipelines of the aircraft and engine nozzles [8], affecting the normal navigation of the aircraft and creating a major safety hazard.

Heat transfer coking of Chinese RP-3 is a complex process. Owing to the different operating temperatures, the main types of chemical reactions that occur during fuel heat transfer and the composition and morphology of the deposits produced by their heat transfer can vary considerably [9,10]. Compared with C–C bond breaking, which requires a higher activation energy, oxygen’s reaction with hydrocarbon molecules requires less activation energy to generate alkoxy radicals [11,12]. Therefore, the fuel can be oxidized and deposited at a lower temperature, whereas the cracking reaction requires a higher starting temperature. It is now commonly believed that the fuel undergoes mainly oxidation reactions to produce colloid deposits in the temperature range from 150 °C to 370 °C [13]. At temperatures above 480 °C, the fuel produces deposits mainly by cracking reactions [14]. A large number of researchers have investigated the effects of changes in fuel composition [15,16], dissolved oxygen content [17,18,19], metal surface catalysis [20], fuel polar substances [21,22,23], and fuel flow patterns [24,25,26,27] on the thermal deposition of fuels. The heat transfer deposition of RP-3 includes not only thermal oxidation deposition generated at lower temperatures, but also the thermal cracking deposition generated at higher temperatures. The chemical reaction paths and products of fuel in different temperature ranges are quite different, and the macro structure and micro composition of the deposits are also quite different. It is generally accepted that as the fuel flows and gets heated up, dissolved oxygen will react with hydrocarbons to form compounds containing carboxyl, hydroxyl, and other oxygenated groups. These molecules have a higher polarity and reactivity compared to non-oxidized hydrocarbons, and thus are more likely to precipitate or polymerize in the continuous phase of fuels to produce macromolecular deposits [15].

In this work, we experimentally investigated the deposition behavior of RP-3 after heat transfer in a unidirectional heating tube, and examined the changes in the amount of oxidation deposition generated during the heat transfer of the fuel using dissolved oxygen content (DOC) as a variable. In situ analysis of the trace coke mass in the reaction tube was also carried out using Raman spectroscopy, and the structure of the formed coke mass was analyzed in detail by combining SEM with microscopic observation of the internal surface structure of the reaction tube. The carbon content of the micro coke produced was analyzed using the temperature-programmed oxidation method. Explaining the heat transfer deposition mode of fuel in detail can lead to a better understanding of the fuel deposition behavior in the oxidation–cracking coupling temperature region. The gradual accumulation of surface deposition during the long-term operation of aeroengine air-fuel heat transfer can largely alter the morphology and properties of the reaction interface, eventually leading to significant changes in the deposits. Therefore, it is of great significance to study the structural properties and mode of formation of the deposits produced by RP-3 in the process of heat transfer.

## 2. Results

In this paper, fuel deposition behavior under different dissolved oxygen concentrations and temperatures was investigated by fuel heat transfer experiments. A combined study of microscopic observation and analysis on the film structure of deposits was conducted to analyze the behavior of deposits generated by oxidation and cracking reactions of RP-3 aviation kerosene during heat transfer. 

The quantitative oxidation analysis showed that the high-temperature fuel which flowed by microchannels formed deposits on the metal surface. There was a correla-tion between the mass of deposits formed and the DOC in the fuel, indicating that the main source of deposition formed by the fuel was derived from thermal oxidation. The mass, structure, and morphology of the deposits on the tube’s surface would significantly change under higher temperatures or over longer reaction times. When the reaction temperature reached 527 °C, there were a lot of deposits at the end of the reaction tube after heat transfer, which was vastly different from the deposits formed at 437 °C.

### 2.1. Morphology of Deposits

#### 2.1.1. Differences in the Internal Surface of a Reaction Tube after Heat Transfer of Aviation Kerosene with High DOC

**Figure 1:**
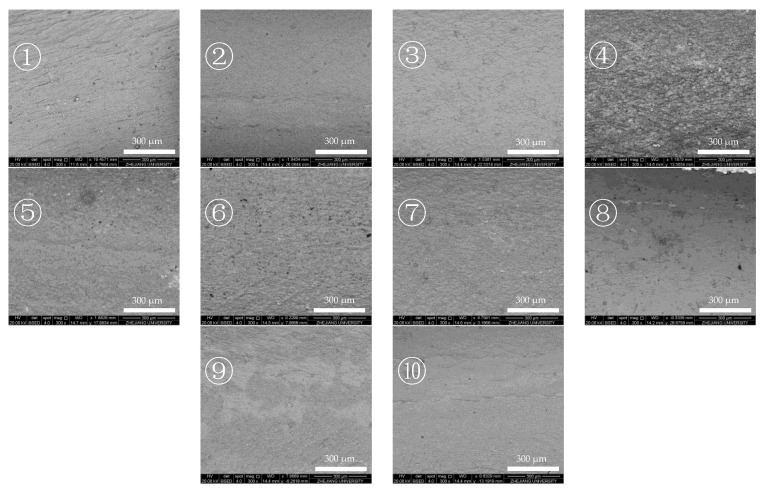
BSED images of the internal surface of the reaction tube after heat transfer of aviation kerosene with a dissolved oxygen content (DOC) of 41.2 ppm. The number “①” to ”⑩” in the figures represent a reaction tube with an average of 10 sections, with directions from “①” to ”⑩” representing the direction of fuel flow in the experiment.

Figure 1 shows the images of the internal surface of each section of the reaction tube in a large range. The darker part is the aggregation area for elements with low atomic numbers. Considering that the reaction tube material is mainly metal with a high atomic number, the darker part is mainly the deposits on the internal surface of the reaction tube. The figure shows that there is a large dark area in section ④ of the reaction tube, which can be ascribed to C and O accumulation as opposed to the area with metal oxides. The BSED images are in general agreement with the trend data on deposit contents obtained from Raman spectroscopy, with deposits containing carbon in the front and middle sections of the reaction tube, and relatively little deposit formed in the inlet and rear sections of the reaction tube under this heat transfer condition.

**Figure 2:**
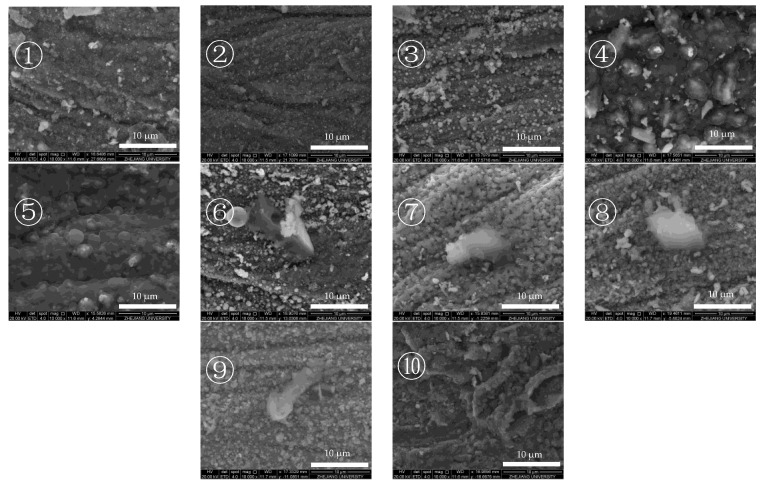
ETD images of the internal surface of the reaction tube after heat transfer of aviation kerosene with a DOC of 41.2 ppm. The number “①” to ”⑩” in the figures represent a reaction tube with an average of 10 sections, with directions from “①” to ”⑩” representing the direction of fuel flow in the experiment.

**Figure 3:**
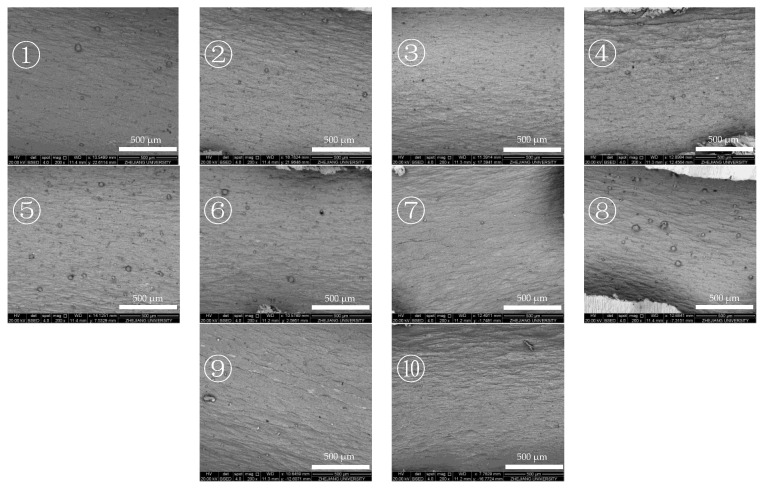
BSED image of the internal surface of the reaction tube after heat transfer of aviation kerosene with a DOC of 2.7 ppm. The number “①” to ”⑩” in the figures represent a reaction tube with an average of 10 sections, with directions from “①” to ”⑩” representing the direction of fuel flow in the experiment.

**Figure 4:**
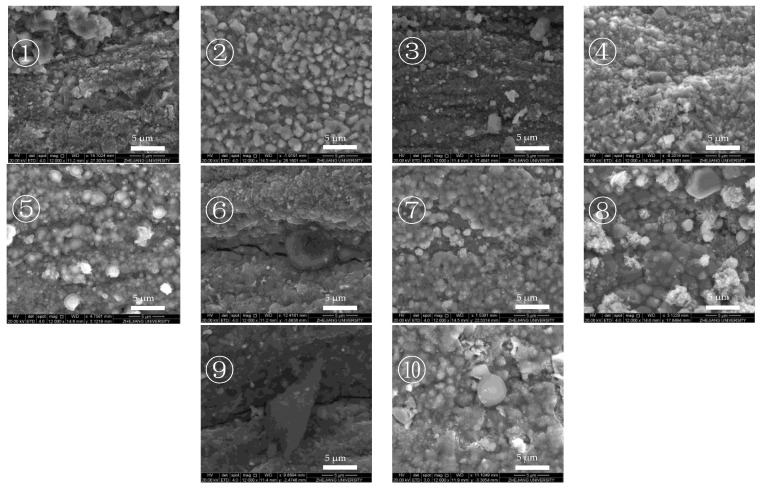
ETD images of the internal surface of the reaction tube after heat transfer of aviation kerosene with a DOC of 2.7 ppm. The number “①” to ”⑩” in the figures represent a reaction tube with an average of 10 sections, with directions from “①” to ”⑩” representing the direction of fuel flow in the experiment.

Figure 2 shows a magnified ETD image of the area in Figure 3. ETD images enable a clearer view of the deposits’ morphology inside the reaction tube. The images of the reaction tube segments in Figure 3 and Figure 4 reveal that the internal surface of the reaction tube was distributed with an axial groove-like texture. Raised polygonal particles with clear edges are also visible on the internal surface of some of the reaction tube segments, and the higher atomic lining of such particles indicates that they are metal oxide crystals and are not continuously covered by deposits. In the images of section ④ and ⑤ of the reaction tube, the axial texture of the metal internal surface is completely invisible, and the metal oxide crystals with clear edges have also become amorphous bumps with blurred edges. Overall, the deposits are distributed in a laminar cover in the internal surface of the reaction tube. The remaining pictures show the irregular granular distribution of deposits along the tube’s internal surface after fuel heat transfer.

#### 2.1.2. Differences in Internal Surface of the Reaction Tube after Heat Transfer of Aviation Kerosene with Low DOC

Figure 3 shows the BSED image of the internal surface of the reaction tube after 100 min of heat transfer with a low-DOC fuel at an outlet fuel temperature of 437 °C, flow rate of 1.0 g/s, and pressure of 4.0 MPa. In a large range of BSED images, it can be seen that the internal surface of the reaction tube appears as a metal atom background with less scattered dark spots, and there is no densely covered dark area, such as those that can be observed in Figure 1. This indicates that the deposits’ distribution in this reaction tube is more dispersed and the total amount of deposits is relatively low.

Figure 4 shows the image of the internal surface of the reaction tube after the heat transfer of a fuel with a low DOC. The effect of the heat transfer of the deoxygenated fuel on the internal surface of the reactor tube is small, the edges of the metal oxide particles are clear, and the inner surface of the reactor tube is not covered. Only granular deposits can be observed inside the defects of the tube’s surface. It is considered that these defects generally have a higher specific surface area and are thus more likely to trap macromolecules. Thus, deposits are mostly observed in these defects [28].

#### 2.1.3. Element Analysis of the Internal Surface of the Reaction Tube

**Figure 5:**
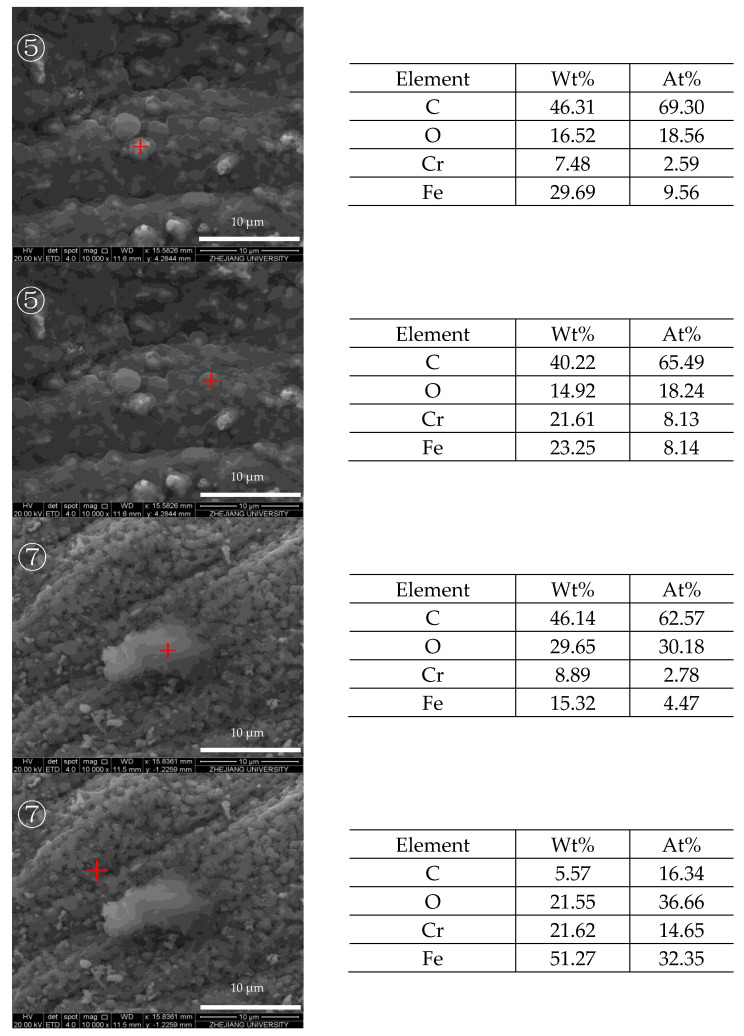
Morphology of the internal surfaces of reaction tubes ⑤ and ⑦, and elemental analysis of the selected corresponding area (marked with red “+” in the images).

The elemental analysis of the reaction tube internal surface is shown in Figure 5. In the image of reaction tube section ⑤, the selected areas of the image are all rich in carbon, yet have little oxygen, indicating the deposits are mainly formed by carbonous coke instead of oxygen-rich gums. The elemental analysis of the granular deposits in tube ⑦ shows that the exposed metal oxides have almost no carbon, whereas the surrounding area of granule deposits is rich in carbon, confirming the presence of deposits in the area.

After the heat transfer of fuel with a DOC of 41.2 ppm, the internal surface in the front and middle sections of the reaction tube are covered by a deposition layer, whose elements on the surface are mainly carbon and oxygen instead of iron and chromium on the surface of the bare reaction tube. However, after the heat transfer of fuel with low DOC, no such deposition layer is visible on the internal surface of the reaction tube. In the supercritical heat transfer experiment of RP-3 in this work, the DOC of the fuel affected the form of deposits. When the DOC of the fuel was high, the concentration of polar oxidation products in the fuel increased rapidly after heating up. The higher the concentration of oxidation products, the easier it was for them to adsorb with the internal surface of the reaction tube or agglomerate with each other. According to the results of elemental analysis, the carbon content in the layered deposits was relatively higher, while the oxygen content of granular deposits was higher. This may indicate that the layered deposits adhered to the internal surface of the reaction tube for a longer time, resulting in more heat transfer and further aggregation of macromolecular polar substances. The presence of oxygen on the surface of the varied deposits also indicated that the DOC of fuel affects the amount of deposits during supercritical fuel flow.

The above experiments verify that the heat transfer of fuel at a flow rate of 1.0 g/s at 437 °C and 4.0 MPa can produce different amounts of deposits in the metal tube wall, and the amount of deposits is related to the DOC in the fuel.

### 2.2. Raman Spectroscopy of Deposits Structure

**Figure 6:**
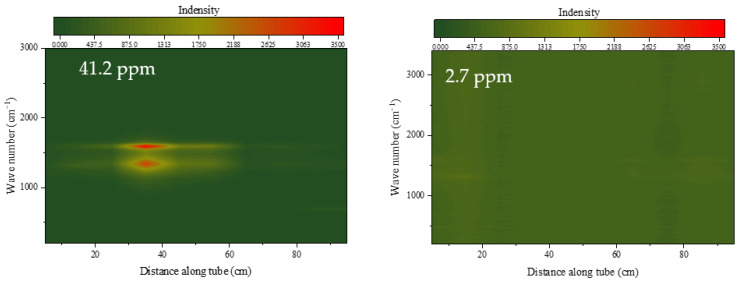
Raman spectra of tube surface after heat transfer by RP-3 with different DOCs.

Figure 6 shows the Raman spectra of the internal surface of the heat transfer reaction tube after continuous operation by RP-3 with different DOCs for 100 min at a flow rate of 1.0 g/s, an outlet fuel temperature of 437 °C, and a pressure of 4.0 MPa.

Figure 6 shows the diffraction results of the Raman spectra of the inner surface of the heat transfer reaction tube. The same color scale is used in the left and right figures. When the DOC of the fuel is high, a more obvious Raman absorption double peak can be observed in the front section of the reaction tube at 1350 cm^−1^ and 1590 cm^−1^, respectively. The peak at 1590 cm^−1^ indicates that the sample surface contains a typical aromatic hydrocarbon structure, the peak at 1350 cm^−1^ is a breathing mode of six-fold carbon rings which only becomes active in the presence of disorder carbon [29]. Fuel with a higher DOC will leave very obvious peaks in the middle section of the reaction tube, but fuel with a lower DOC has peaks brought by aromatic hydrocarbons that are difficult to see. At the same time, there is a more obvious Raman peak appeared at 690 cm^−1^ in the first section of the reaction tube, which is the typical absorption peak of Fe_3_O_4_.

According to previous elemental analysis results, there are metal oxides on the inner wall of the reaction tube, which are also reflected in the Raman spectroscopy results. The microscopic morphology and elemental analysis show distinctive differences, which are reflected in the morphology and element content ratio of the deposits distributed along the reaction tube. These results correspond to the results of Raman spectroscopy. It can be observed that all test reaction tubes contain a concentrated area of deposits, and the structure and morphology of the deposits in this area are significantly different from those of the rest of the tube.

### 2.3. Quantitative Analysis of Deposition

**Figure 7:**
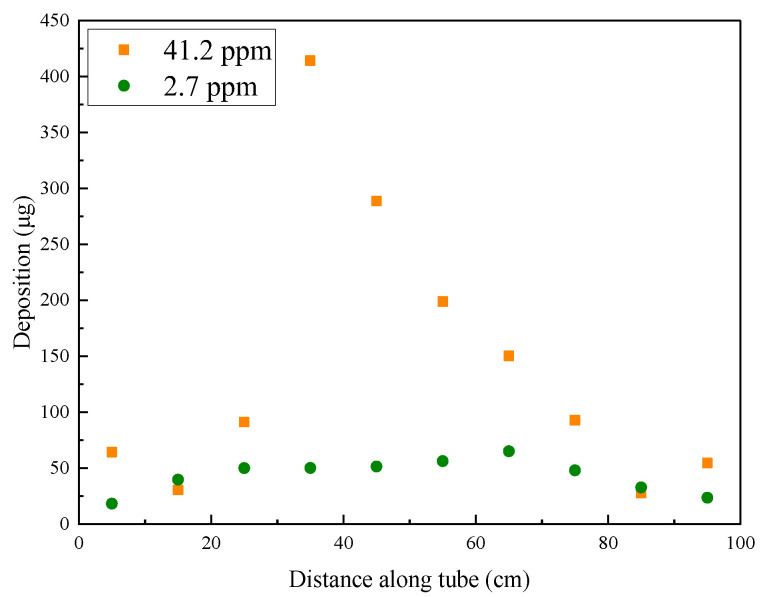
Mass of carbon deposited across the reaction tube after heat transfer by RP-3 with different DOCs.

According to the analysis of the material properties of the internal surface of the reaction tube after heat transfer, RP-3 will produce deposits with carbon as the main component under the conditions of flow and heat transfer. Figure 7 shows that the mass distribution of elemental carbon in the deposits obtained by the temperature-programmed oxidation method is consistent with the SEM observation results. Specifically, fuels with a higher DOC formed a distinct area of elemental carbon deposition aggregation in the middle section of the reaction tube, whereas the deposit amounts of fuels with a lower DOC in the reaction tube were low. The mass distribution of the deposits indicates that the thermal oxidation deposition of fuels influenced by the DOC is concentrated in the middle section of the reaction tube under experimental conditions.

**Figure 8:**
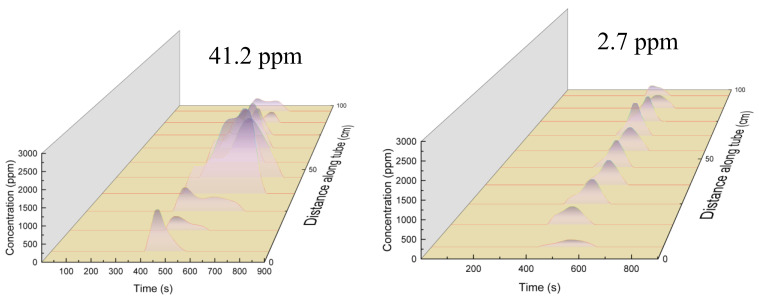
Release rate of CO_2_ in different tube sections during temperature-programmed oxidation.

Figure 8 shows the release rate of CO_2_ during the oxidation of deposits in the reaction tube after heat transfer by RP-3 with different DOCs. The curves in the figure represent the variation in CO_2_ concentration released with time during the oxidation of the deposits at different locations in the segments of the reaction tube. The oxidative deposits in the low-DOC fuels generally show a single-peak curve, whereas the decomposition curves of deposits generated by the high-DOC fuels mostly have hysteresis peaks, especially in the 25 cm to 55 cm section. In contrast to the oxidative decomposition curves of the high-DOC fuel deposition, the single-peaked curve of the low-DOC fuels indicates that the deposition oxidizes and decomposes at a relatively constant rate, while the tailing peak of the deposition produced by the high-DOC fuels indicates that they have higher thermal stability components and their oxidative decomposition rate is slower [30]. Compared with granular deposits, the layered deposits generated by high DOC fuels are denser and more thermally stable.

### 2.4. Analysis of Deposition Structure Produced at Higher Temperature

In previous experiments, we observed differences in deposit morphology on the surface of the reaction tube near the outlet, so we speculate that the temperature of the reaction tube will have an impact on the deposition of fuel transfer. To compare the deposition differences caused by different temperatures in the heat transfer process of RP-3, an experiment was conducted by increasing the reaction temperature and reaction time in fuel heat transfer to ensure that the oxidation reaction and cracking reaction could produce enough of an observed deposition. The pressure of the device remains at 4.0 MPa, and the fuel flow rate is reduced to 0.2 g/s, and a heat transfer simulation experiment is conducted at outlet fuel temperatures of 437 °C and 527 °C for a running time of 100 min. The DOC of the fuel is 9.2 ppm.

**Figure 9:**
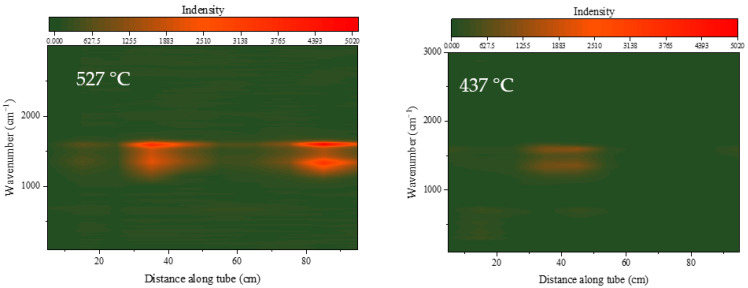
Raman spectra of the tube surface at an outlet fuel temperature of 527 °C (flow rate = 0.2 g/s, pressure = 4.0 MPa, running time = 100 min).

As is shown in Figure 9, when the outlet fuel temperature is 527 °C, the deposition generated on the internal surface of the reaction tube during the fuel heat transfer process has peaks at 1350 cm^−1^ and 1590 cm^−1^. At the same time, weaker peaks can be observed below 1000 cm^−1^, especially at 690 cm^−1^, which is the typical peak of Fe_3_O_4_. Due to the surface of the sample being covered by deposits generated after fuel heat exchange, the signal strength of the metal oxide is relatively weak. The peak of Fe_3_O_4_ on the reaction tube at the inlet is relatively strong, and as the temperature of the tube wall increased and deposits were generated, the peak intensity here also weakened. The peak of Fe_3_O_4_ at 60 cm of the reaction tube is relatively obvious, indicating that there is iron oxide exposed on the surface. This is consistent with our speculation about the formation mode of sediment at this location: after the concentration of oxidation products decreases, the coverage of oxidation products in the fuel on the inner wall of the reaction tube decreases. In the same situation, the peak at 690 cm^−1^ can be seen relatively more clearly on the inner wall of the reaction tube with a heat exchange temperature of 437 °C.

**Figure 10:**
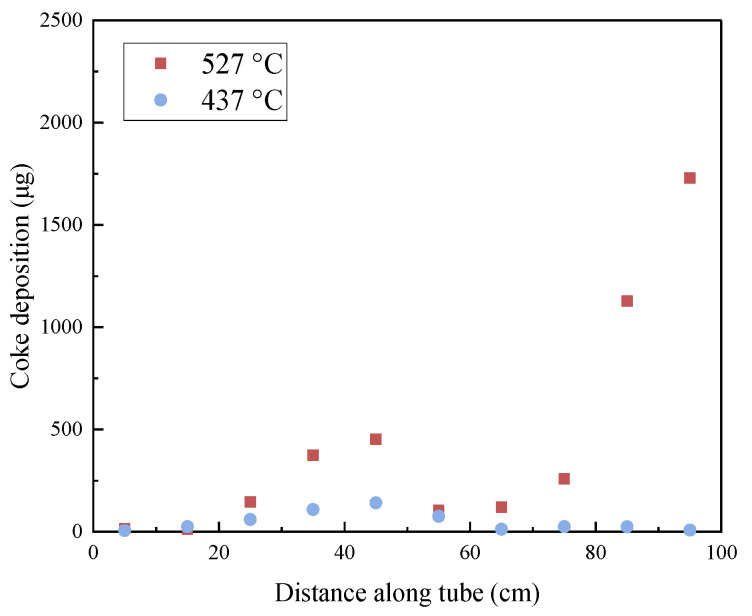
Mass of carbon deposited at different outlet fuel temperatures (flow rate = 0.2 g/s, pressure = 4.0 MPa, running time = 100 min).

As shown in Figure 10, the mass distribution of deposits in the internal surface section of the reaction tube after heat exchange at an outlet fuel temperature of 527 °C is different from that of the deposits after heat transfer at an outlet fuel temperature of 437 °C. The difference is that there are more deposits at the outlet end of the reaction tube, and the generation of such deposition may be different from that of the middle section. The deposition in the low-temperature area of the middle section of the reaction tube is mainly caused by oxidation, so the change in DOC will affect heat transfer deposition in this area. At the end of the reaction tube, after the outlet fuel temperature reaches 527 °C, the aviation kerosene will also undergo more violent cracking reactions, dehydrogenation of hydrocarbon molecules, carbonization, and other changes. The large amount of deposits at the outlet of the reaction tube is less affected by the DOC of fuels.

**Figure 11:**
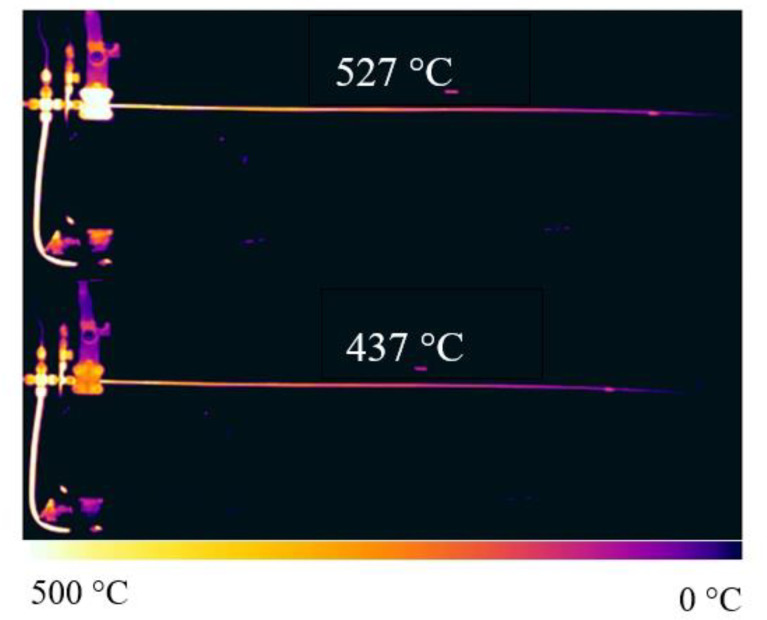
Infrared thermographic image of the reaction tube during heat transfer of aviation kerosene at different outlet fuel temperatures (flow rate = 0.2 g/s, pressure = 4.0 MPa).

**Figure 12:**
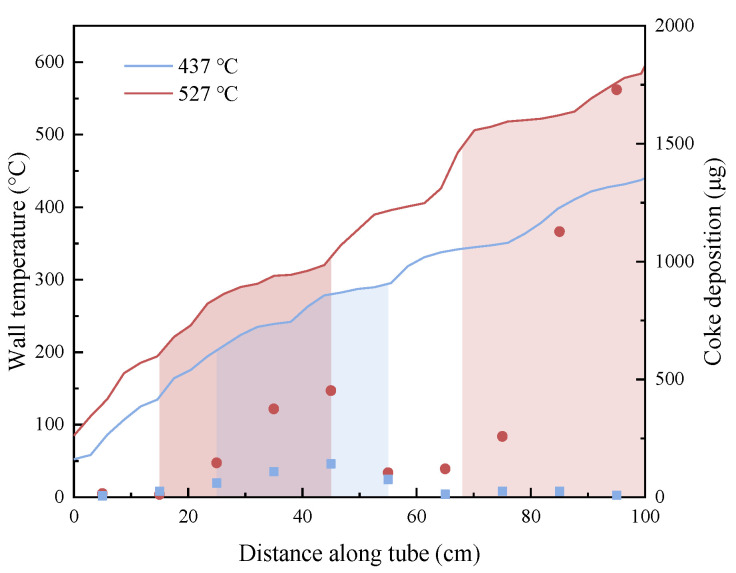
Temperature distribution of the reaction tube monitored by thermal camera.

Figure 11 and Figure 12 show the combined image of the outer wall temperature distribution and deposition distribution of the reaction tube when the outlet fuel temperature is different. Figure 12 shows the wall temperature along the outer wall of the reaction tube after the image signal in Figure 11 is converted into a digital signal, and the marked color area is the distribution area of the deposition produced on the internal surface of the corresponding reaction tube. It can be seen from the figure that the deposition caused by oxidation occurs in the external wall temperature range of 200~300 °C. The corresponding 300~400 °C area did not produce obvious deposition. As the outer wall temperature continues to rise to more than 500 °C, the reaction tube with an outlet fuel temperature of 527 °C had obvious coking in this area, while the reaction tube with an outlet fuel temperature of 437 °C had almost no coking in this area.

For fuels with the same DOC, the heat transfer temperature had a more significant impact on the deposition. A large amount of deposits were generated in the area where the reaction tube temperature exceeded 500 °C, which were much more in mass than the deposits generated in the middle section of the reaction tube. Although the deposit amount in the middle section of the reaction tube increased slightly when the outlet fuel temperature increased from 437 °C to 527 °C, this was only due to the increase in average temperature. The deposits generated at the end of the reaction tube are directly related to the increase in temperature of the tube, and there may be significant differences between them and the deposition caused by dissolved oxygen in the fuel.

**Figure 13:**
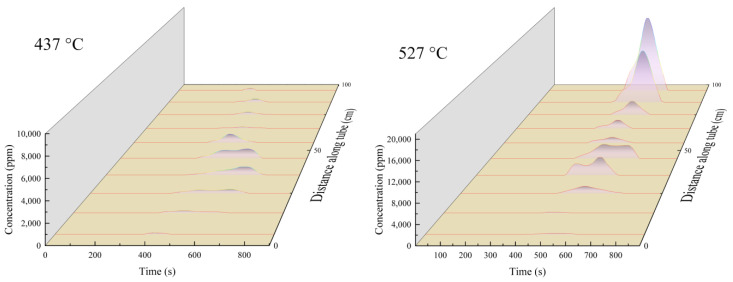
Release rate of CO_2_ in different tube sections during temperature-programmed oxidation.

Figure 13 shows the comparison of the concentrations of CO_2_ released by the oxidation of deposits in the reaction tube after heat transfer at different outlet fuel temperatures. It can be seen that in the reaction tube with an outlet fuel temperature of 527 °C, the oxidative decomposition of deposits in the front and middle sections presented a bimodal pattern. The oxidative decomposition of a large amount of deposition at the end of the reaction tube released CO_2_ at a uniform rate. This indicates that when the heat transfer temperature increased, the fuel generated more deposits, and that the microstructure of deposits was different: the deposition near the end of the reaction tube was more prone to oxidative decomposition, and was not as compact as the deposition produced by the oxidation reaction in the stacking mode.

According to the distribution of deposition at different outlet fuel temperatures, two deposition areas can be observed when the outlet fuel temperature reached 527 °C. Therefore, SEM is used to observe the morphology of deposition in different areas of the reaction tube at an outlet fuel temperature of 527 °C in two modes.

**Figure 14:**
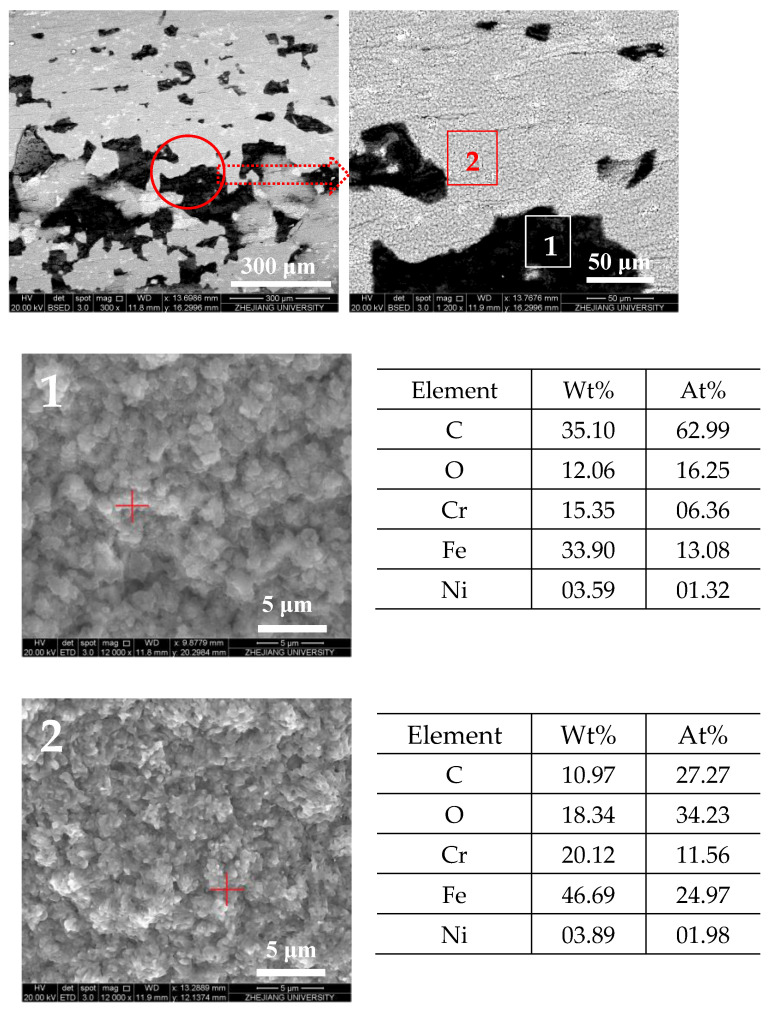
Morphology of the reaction tube surface in the oxidation deposition area and elemental analysis of the selected corresponding area (marked with red “+” in images) (outlet fuel temperature = 527 °C, flow rate = 0.2 g/s, pressure = 4.0 MPa, running time = 100 min). (**1**) Enlarged image of the area marked “1” in the right image. (**2**) Enlarged image of the area marked “2” in the right image.

**Figure 15:**
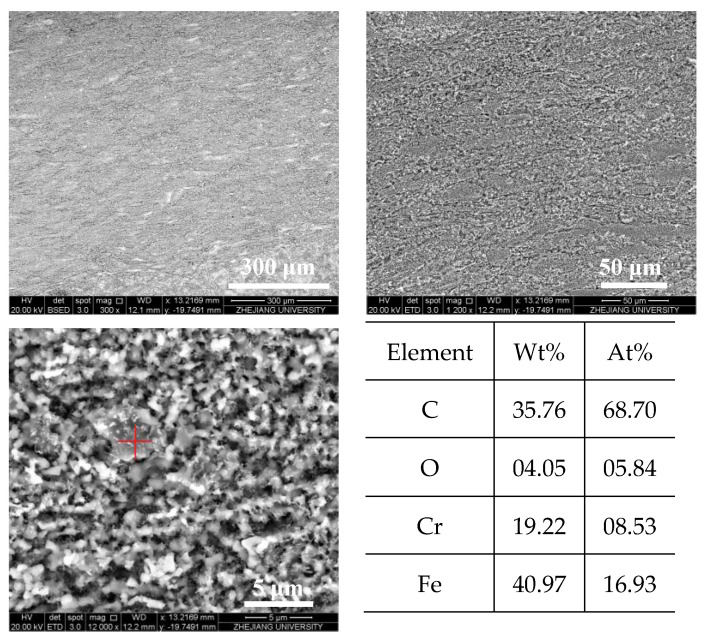
Morphology of the reaction tube surface in the cracking deposition area and elemental analysis of the selected corresponding area (marked with red “+” in images) (outlet fuel temperature = 527 °C, flow rate = 0.2 g/s, pressure = 4.0 MPa, running time = 100 min).

Figure 14 and Figure 15 show the SEM images of the internal surface of the reaction tube in different areas at an outlet fuel temperature of 527 °C. It can be clearly observed in the figures that under the broad view of BSED, the oxidation deposition area is covered by dark deposits, while the cracking deposition area does not show a wide range of dark deposits. However, after the magnification is increased, it can be observed that the pores and defects of the tube’s internal surface in the cracking deposition area have been filled with dark deposits, indicating the presence of carbon and oxygen atoms. As is shown in Figure 14, the element analysis results of different regions in the SEM image indicate that there was an uneven distribution of deposits on the reaction tube in this section; the extent to which the surface of the reaction tube was covered by deposits varies. Further observation of the deposit morphology using the ETD mode shows that the metal oxide particles exhibited irregular edges, and contained more carbon elements. Figure 15 shows the microstructure observation and elemental analysis of the final section of the reaction tube. Except for the significant differences in the microstructure of the surface of the reaction tube, element analysis results on its surface show that the content of oxygen is lower, while containing more carbon, resulting in the deposits having a higher degree of dehydrogenation due to the pyrolysis reaction.

The morphology of these deposits can be attributed to the oxidation reaction between fuel and dissolved oxygen. The oxidation reaction initiated by oxygen-free radicals rapidly produces oxygen-containing hydrocarbons as the fuel temperature increases. These polar oxides are more easily adsorbed onto the reaction tube surface or polymerized into polar macromolecules. Therefore, layered deposits are formed by the continuous adsorption of small-molecule oxides onto the surface. When dissolved oxygen in the fuel is consumed, these layered deposits are not observed on the internal surface of the reaction tube in the rear section. Instead, polar macromolecules that have not been adsorbed onto the surface and polymerize with each other form granular deposits after being adsorbed onto the surface of the reaction tube. The deposition generated in the area with higher temperature was filled with metal oxide crystals and connected with filamentous carbon. According to the experimental results of temperature-programmed oxidation, the deposits at the end of the reaction tube did not cover each other and generated a filamentous structure. Therefore, fuel deposition at high temperatures was more likely to be generated by the metal catalysis of the reaction tube surface. These filamentary cokes are exactly the typical hydrocarbons catalyzed by metals at high temperatures, owing to the upward migration of metals with the growth of filamentary cokes and the formation of indeterminate carbon nanowire structures [31].

From the experimental results of Raman spectroscopy, morphology observation, and temperature-programmed oxidation of the deposits, it can be inferred that the deposition generated by the oxidation reaction is diffused from the fuel to the reaction tube’s surface, while the deposition generated by the pyrolysis reaction at high temperature is in situ generated on the surface of reaction tube.

The heat transfer deposition characteristics of aviation kerosene have always been considered to be related to its oxidation stability, but there are always differences in quantitative analysis on the deposits after fuel heat transfer. It was found that the deposit structure generated by fuel under different reaction conditions is different. It is necessary to understand the thermochemical deposition characteristics of aviation kerosene or higher density endothermic hydrocarbon fuels. The method proposed in this article is not only applicable to the study of heat transfer deposition characteristics of aviation kerosene, but also to the study of high-temperature thermal cracking deposition characteristics of high-density hydrocarbons. The characteristics of temperature distribution and composition distribution of the fuel in the reaction tube can also be calculated by the Raman spectroscopy quantitative analysis of the deposits on the reaction tube’s surface.

## 3. Materials and Methods

### 3.1. Materials

Toluene, acetone, isopropanol, and n-hexane were purchased from Sinopharm Chemical Reagent Co., Ltd. (Shanghai, China), and RP-3 was purchased from Guangdong Zhonghai Nanlian Energy Co., Ltd (Guangzhou, China). All of the reagents were used as received without further purification. The basic properties of the fuel are shown in Table 1. The gas chromatograph mass spectrometer used was the Agilent 7890/5975C (Hangzhou, China) and the components of aviation kerosene are shown in Table S1 of the Supplementary Materials.

### 3.2. Simulating Fuel Flow and Heat Transfer

#### 3.2.1. Preparation of Aviation Kerosene with Different DOCs

The dissolved oxygen content of aviation kerosene was replaced with saturated pure gas, and a total of three fuels with different oxygen contents were prepared. Taking the preparation of aviation kerosene with low oxygen content as an example, aviation kerosene was put into a special container, and the top and bottom of the container were, respectively, equipped with an inlet and outlet for fuel and gas to enter and exhaust, and the switch was controlled by a ball valve. After the nitrogen gas stream (99.9%) passed by the piping, the oxygen was displaced with the use of ceramic bubble diffusers to generate fine bubbles that floated upward. The operational procedure was as follows. First, the oil outlet and oil injection port were closed, the nitrogen cylinder was opened, nitrogen blew in from the air outlet, the exhaust port was opened for exhaust, and the container was purged for 10 min to discharge the air. Then, the nitrogen was closed, the nitrogen atmosphere was maintained in the container, and aviation kerosene was injected into the container by the oil injection port, reserving 5% of the volume. Afterwards, the oil injection port was closed, the nitrogen and upper exhaust ports were opened, and the N_2_ flow rate was set to a fluid ratio of 1.0 between the nitrogen and the oil to be treated for 30 min. The dissolved oxygen content in the aviation kerosene was measured using a dissolved oxygen tester (S4-Meter, Mettler Toledo, Greifensee, Switzerland). After the gas inlet and outlet valves were closed, the oil outlet was opened to keep the sample to be measured in a closed container.

#### 3.2.2. Thermal Deposition of RP-3 Fuel

An electrically heated tubular reactor is used to simulate the heat transfer process of aviation kerosene in microchannels. The fuel flux, system pressure, heating power, and heat transfer duration can be controlled during the operation of the device, and the parameters that can be measured include the fuel outlet temperature, wall temperature of the heat transfer reaction tube, and pressure drop at the front and rear ends of the reaction tube.

**Figure 16:**
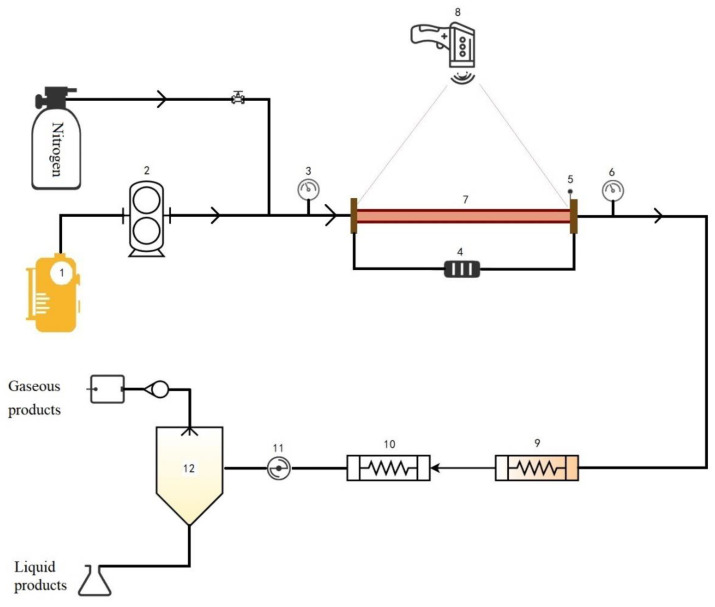
Schematic diagram of the flow heat transfer simulation device: (1) fuel storage tank; (2) high-pressure constant-flow pump; (3) inlet pressure sensor; (4) DC-electric heater; (5) K-type thermocouple; (6) outlet pressure sensor; (7) heat transfer reaction tube; (8) infrared thermometer; (9 and 10) condenser tubes; (11) back-pressure valve; and (12) gas–liquid separator.

The schematic diagram of the fuel flow heat transfer simulator is shown in Figure 16. It includes five parts: a fuel flow control system, an electric heating system, a cooling sample collection system, a gas–liquid separation system, and a data acquisition system. The main equipment of the unit included a Φ4.0 × 1.0 mm GH3128 alloy tube, and the heat transfer reaction tube was a Φ3.0 × 0.5 mm 316 L stainless steel tube. The device could heat hydrocarbon fuels with flow rates from 0.1 to 5.0 g/s and pressures from 0 to 6.0 MPa with a heat flow up to 2 MW·m^−2^, allowing the fuel to reach the target temperature in a short time to simulate heat transfer.

### 3.3. Structural and Morphological Analysis of Deposits on the Internal Surface of Reaction Tubes

After the reaction tubes for simulating flow heat transfer were sectioned, cleaned, and dried with n-hexane (for details, see Supplementary Materials SM1), the tubes were cut axially to expose their inner surfaces. This process requires great care and caution to ensure that localized high temperatures in the tubes do not damage the deposits. A Raman spectrometer (LabRam HR UV, Jobin-Yvon, Paris, France) with a 514 nm-wavelength light source (power of 50 mW with a 5 μm spot diameter) was used to scan the internal surface of each reaction tube, and the morphology of the deposits on the internal surface of the tube was observed using an SEM (FEG650, FEI, Eindhoven, The Netherlands). Back-scattering electron detector (BSED) images and high-vacuum secondary electron transfer dissociation (ETD) images were used to observe the morphology with the SEM. The BSED image had a different color contrast because of the different atomic numbers in the field of vision.

## 4. Conclusions

In this paper, fuel deposition behavior under different dissolved oxygen concentrations and temperatures was investigated by fuel heat transfer experiments. A combined study of microscopic observation and analysis on the film structure of deposits was conducted to analyze the behavior of deposits generated by oxidation and cracking reactions of RP-3 aviation kerosene during heat transfer, and the following conclusions were obtained.

It was found that the DOC in the fuel not only affected the quality but also the morphology of the deposits. After flow experiments, by comparing the decomposition rates of deposits formed at different oxygen contents, it was further found that the DOC also affected the density and composition of deposits.

The mass, structure, and morphology of the deposits on the tube’s surface would significantly change under higher temperatures or over longer reaction times. When the reaction temperature reached 527 °C, there was a lot of coking at the end of the reaction tube after heat transfer. It is identified that the deposits in the middle section of the reaction tube were covered due to oxidation, while the deposits at the end of the reaction tube formed filamentous coke under the catalytic effect of the metal walls. It is further revealed by Raman spectra and temperature-programmed oxidation that the cracking cokes were not as tightly packed as those formed by oxidation. For the heat transfer of aviation kerosene in a certain flow rate range, the morphology and structure of the deposits was related to the temperature of the outer wall of the reaction tube: oxidative deposition mostly occurred at a wall temperature range of 200–300 °C, whereas cracking deposits generally emerged above 400 °C. The deposition generated by the oxidation reaction was diffused from fuel to the reaction tube’s surface, while the deposition generated by the pyrolysis reaction at a high temperature was in situ generated on the surface of reaction tube.

## Figures and Tables

**Table 1:** The basic properties of RP-3 (25 °C, 101 kPa).

Sample Name	RP-3
Denstity (g/cm^3^)	0.811
Dynamic viscosity (mm^2^/s)	2.022
Closed-cup flash point (°C)	44.5
Total acid value (mg KOH/g)	0.0038
Aromatics content (*v*/*v* %)	9.4
Olefin content (*v*/*v* %)	1.4
Dissolved oxygen content at atmospheric conditions (ppm)	9.2
T501 content (mg/L)	21

## Data Availability

Data is contained within supplementary material. The data presented in this study are available in Supplementary Materials.

## Supplementary Materials

The following supporting information can be downloaded at https://www.mdpi.com/article/10.3390/molecules28114508/s1: Table S1, Composition of aviation kerosene; SM.1 Determination of heat transfer deposition of aviation kerosene; Figure S1, Carbon combustion unit; Figure S2, Morphology of the inner walls of reaction tubes ⑤ and ⑦ and elemental analysis of the selected corresponding area.
