# Supplementary material for: Morphological and Compositional Analysis on Thermal Deposition of Supercritical Aviation Kerosene in Micro Channels
Source: Molecules. 2023 Jun 1;28(11):4508. doi: 10.3390/molecules28114508 (PMC10254270; doi:10.3390/molecules28114508)
Supplement: Supplementary file 1 [file molecules-28-04508-s001.zip › molecules-2389515-supplementary.pdf]

Supplementary Materials

Morphological and Compositional Analysis on Thermal Deposition of Supercritical Aviation Kerosene in Micro Channels

Ao Sun [1,2], Cui Ye [2], Chenyang Yao [2], Lifeng Zhang [3], Ji Mi [2,*] and Wenjun Fang [2,4,*]

[1] School of Petroleum Engineering, Northeast Petroleum University, Daqing 163318, China; sunaonepu@hotmail.com
[2] Department of Chemistry, Zhejiang University, Hangzhou 310058, China; 22037070@zju.edu.cn (C.Y.); 22037068@zju.edu.cn (C.Y.)
[3] CenerTech Tianjin Chemical Research and Design Institute Co., Ltd., Tianjin 300131, China; zlifeng@zju.edu.cn
[4] Center of Chemistry for Frontier Technologies, Zhejiang University, Hangzhou 310058, China
* Correspondence: 11837037@zju.edu.cn (J.M.); fwjun@zju.edu.cn (W.F.)

Table S1. Composition of aviation kerosene

(Only components exceeding 0.1% are listed, the total number of components is 139.)

Components	Mass fraction(wt%)
Dodecane	4.3935
Tridecane	3.924
Tetradecane	3.4769
cis, cis-3-Ethylbicyclo[4.4.0]decane	3.2388
Undecane, 2,6-dimethyl-	2.8366
Benzene, 2-butenyl-	2.5218
1H-Indene, 2,3-dihydro-4,7-dimethyl-	2.5029
Undecane	2.1665
trans-Decalin, 2-methyl-	2.0614
Nonane, 3-methyl-	2.0463
Decane	1.8918
Bicyclo[3.3.1]nonane	1.8829
Dodecane, 2-methyl-	1.8583
Cyclohexene, 1-butyl-	1.834
Bicyclo[3.1.1]heptan-3-one, 2,6,6-trimethyl-, (1.alpha.,2.alpha.,5.alpha.)-	1.682
cis-Decalin, 2-syn-methyl-	1.6541
Cyclohexane, pentyl-	1.6507
Nonadecane, 9-methyl-	1.6444

Cyclohexane, 1-ethyl-1-methyl-	1.5101
Nonane, 2-methyl-	1.3207
Tridecane, 2-methyl-	1.2747
Cyclohexene,1-(2-methylpropyl)-	1.2265
Ethanol, 2-(dodecyloxy)-	1.1116
Cyclohexane, 1-methyl-2-propyl-	1.1105
Heptadecane, 2,6,10,14-tetramethyl-	1.1056
Decane, 3-methyl-	1.0806
cis-1-Ethyl-3-methyl-cyclohexane	1.0597
Decane, 4-methyl-	1.0449
Naphthalene, decahydro-	1.0225
Cyclohexane, 1,2-dimethyl-3-pentyl-4-propyl-	1.0216

SM.1 Determination of heat transfer deposition of aviation kerosene

Aviation kerosene has a more obvious coking phenomenon in the heat transfer process, and analysis of deposition helps to understand the coking characteristics of the fuel. In this study, the coking in the reaction tube was measured using the combustion carbon method, which was chosen to analyze the carbon content in the deposition relative to the combustion in the reaction tube. The reaction line was sectioned and the coke was oxidized to CO and CO_2 under a high-temperature oxygen atmosphere, and the coke mass was deduced by measuring the contents of the two gases using an infrared analyzer. In this study, we used the carbon combustion method to quantify the conversion of all coking in the reaction tube to CO and CO_2.

A schematic diagram of the carbon combustion unit is shown in Figure 2. The unit is a tube furnace with a pure oxygen source and programmed temperature rise, including an oxygen supply unit, sample oxidation bin, gas secondary oxidation bin, and gas infrared analyzer.

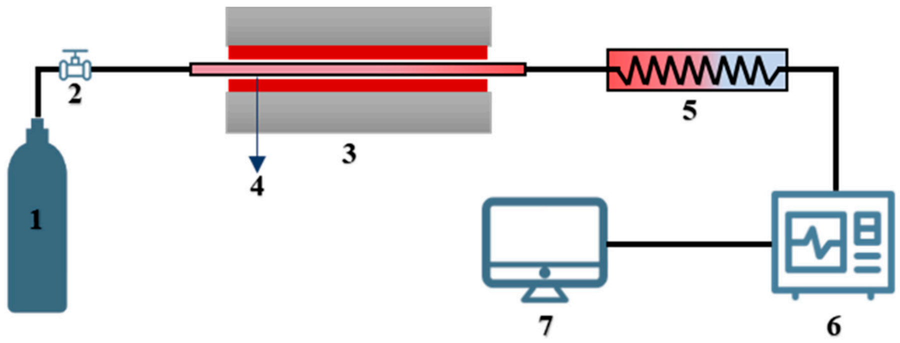

Figure S1. Carbon combustion unit

(1) oxygen source; (2) gas flow meter; (3) tubular heating furnace; (4) sample oxidation bin; (5) secondary oxidation silo; (6) online infrared gas analyzer; (7) data recording system.

The procedure was as follows:

Stage 1. Reaction tube pretreatment. The reaction tube was removed from the flow heat transfer simulator after the simulation, cut into small sections of the same length according to the length of the reaction tube, and each section number was marked according to the position from the inlet end to the outlet end of the heat transfer process. The marked reaction tubes were put into a 250-mL beaker, and 250 mL of *n*-hexane was poured into the beaker. The beaker was sealed with sealing film to prevent the volatilization of *n*-hexane and ensure that the cut reaction tubes could be completely immersed in *n*-hexane during the soaking process. After soaking for 120 min, the reaction tube was taken out, and the small sections of each reaction tube were put in a vacuum oven to dry at 70 °C for 4 h.

Stage 2. Carbon combustion unit preheating. The carbon combustion device was connected according to the diagram, and the gas tightness of the instrument was checked. The oxygen valve was opened, the flow meter was adjusted to 0.5 L/min, and the pipeline was purged for 300 s. The heater was turned on, and the sample bin heater was set to ramp up to 650 °C at 40 °C/min, and the final temperature was held for 300 s. The secondary oxidation section heater was set to heat at 100 °C/min to a constant temperature of 750 °C. After the sample bin heater had been programmed to heat up, the sample bin was opened and cooled down to 250 °C, and then kept at a constant temperature to keep the temperature of the secondary oxidation section heater constant.

Stage 3. Carbon combustion experiments. The completely dried reaction tube was removed, and the sample section to be tested was placed into an oven. The instrument was connected, the oxygen valve was opened to a flow rate of 0.5 L/min, and the pipeline was

purged for 300 s. The sample bin heater was turned on and set to heat at 40 °C/min to 650 °C, which was held for 300 s. The data acquisition system was used to record the CO and CO_2 gas concentrations each second from the start of the temperature rise in the sample bin heater to the end of the time period in which the temperature was held constant at 650 °C.

The deposited elemental carbon mass of the measured sample segment was calculated through the equation:

$$m = \frac{M_{C} p V \int_{t_2}^{t_1} (\mathrm{CO} + \mathrm{CO_2}) \mathrm{d}t}{RT} \times 10^6 \quad \text{Eq.(S.1)}$$

where M_C is the molar mass of carbon (12 g/mol), R is the gas constant (8.314 J·mol^{-1}·K^{-1}), p is the reaction pressure (Pa), V is the flow rate of oxygen (L/s), $\int_{t_2}^{t_1} (\mathrm{CO} + \mathrm{CO_2}) \mathrm{d}t$ is the integrated mass fraction of CO and CO_2 (ppm·s), and T is room temperature (298 K).

Supplement to element analysis results of Figure 7.

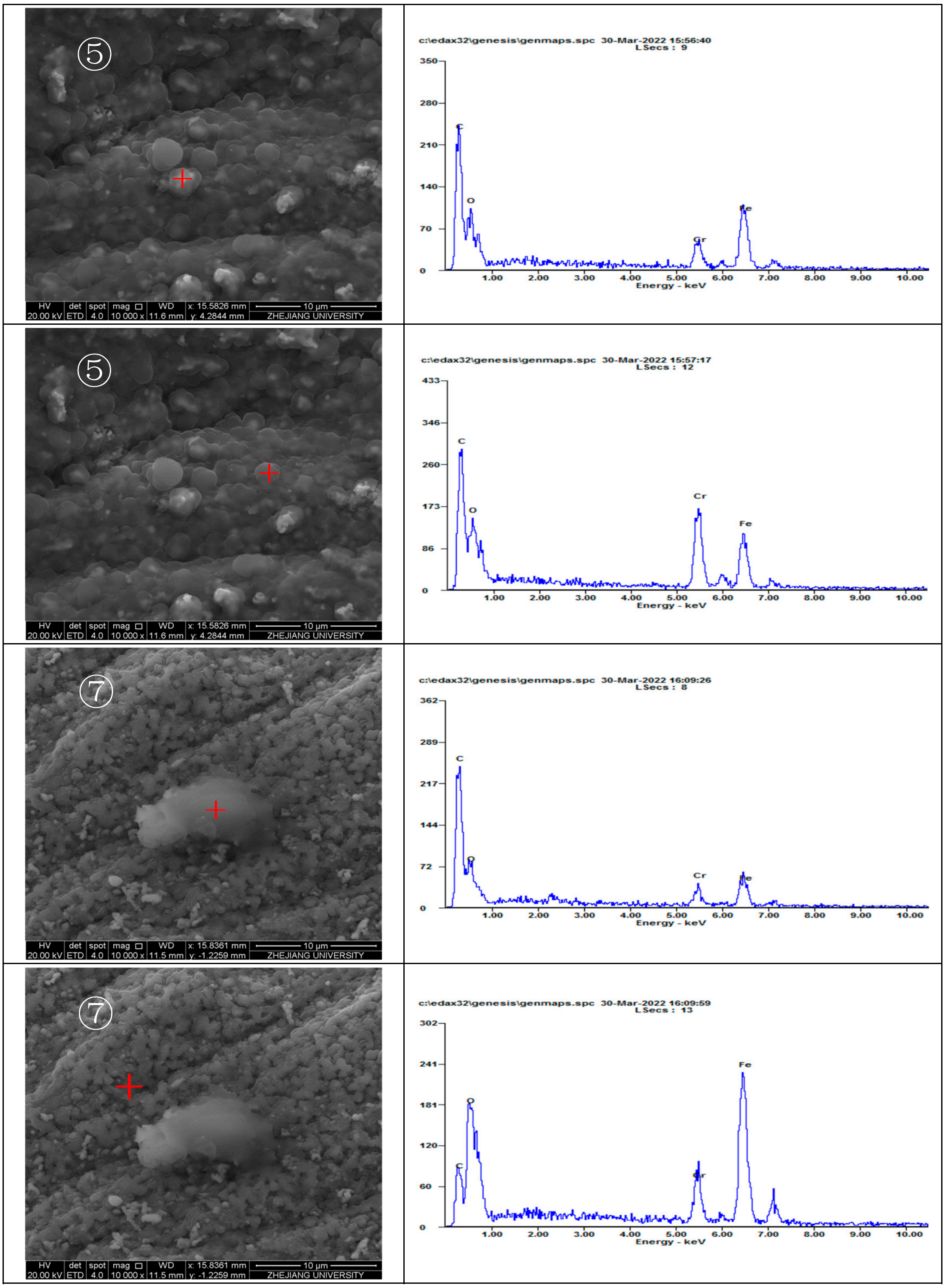

Figure S2. Morphology of the inner walls of reaction tubes ⑤ and ⑦ and elemental analysis of the selected corresponding area.
